# Mental Health Staff's Views on Social Media Use Among People with Psychosis: A Cross-Sectional Survey

**DOI:** 10.1177/20552076251321059

**Published:** 2025-03-17

**Authors:** Xiaolong Zhang, Natalie Berry, Sandra Bucci

**Affiliations:** 1 Division of Psychology and Mental Health, School of Health Sciences, Faculty of Biology, Medicine and Health, Manchester Academic Health Science Centre, 5292 The University of Manchester, Manchester, UK; 2 9022 Greater Manchester Mental Health NHS Foundation Trust, Manchester, UK

**Keywords:** Social media, psychosis, staff views, survey, digital, mhealth, ehealth

## Abstract

**Objectives:**

The use of social media is prevalent in society; however, existing evidence is not sufficient to conclude whether the benefits of social media use can outweigh the risks for people with psychosis. In response to a recent call for staff to take a more active role in asking and advising service users about the impact of using social media platforms on their mental health in clinical practice, we sought to understand staff's attitudes toward service users with psychosis using social media in day-to-day life.

**Methods:**

A cross-sectional survey was disseminated from April 2018 to September 2020 in Mental Health Trusts in the Northwest of England.

**Results:**

A total of 155 staff completed the survey. We found that staff's social media use was high; however, as expected, social media was less used for communicating between staff and service users than other digital technologies (i.e., email and text messages). Moreover, staff's awareness of NHS Trust guidelines around communicating with service users via digital technology was limited.

**Conclusion:**

Despite staff views towards service user engagement with social media being mixed, as both benefits and concerns were reported, we need to ensure staff do not overestimate its risks or underestimate its benefits, so that they can offer tailored advice on social media use based on service users’ individual circumstances.

## Introduction

The use of social media is prevalent in society. In 2019, social media platforms were used by more than two-thirds of all Internet users worldwide.^1^ Evidence suggests that the usage of social media in people with psychosis is comparable to the general population.^2–4^ A recent survey showed that over 85% of people with psychosis reported using social media at some point, and nearly half (48%) of those using social media would like to be part of a social media group with other people with psychosis.^5^ In the UK, in response to healthcare services’ increasing usage of social media and other technologies to communicate with service users, NHS England provides guidelines to outline the benefits and risks, as well as confidentiality, safety, ethical, and legal considerations.^6,7^ For example, to maintain professional conduct, staff should refrain from accepting friendship requests from current or former service users on their private or personal accounts.

However, whether the benefits of using social media outweigh the harms in people with psychosis remains unclear.^8^ The main benefits of using social media include facilitating social interactions,^9,10^ accessing peer support networks,^11,12^ and promoting engagement and retention in mental health services,^13,14^ whereas negative effects of social media use include a potential exacerbation of symptoms, such as paranoia,^15,16^ facing hostile interactions via cyberbullying,^17,18^ and risks of disclosing personal health information.^10,19^ Additionally, whilst social media platforms are being explored as tools for detecting and preventing suicide, some users have used social media to announce suicide attempts or even live-stream such acts, highlighting the dual role of social media being used to both potentially prevent or announce suicide behaviour.^20,21^ There has been a recent call for staff to take a more active role in asking and supporting service users about the impact of using social media platforms on their mental health in clinical practice.^22^ In response to this, we sought to understand: i) staff's use of social media platforms; ii)their estimation of service user's use of social media platforms; iii) their communications with service users via social media platforms and other digital tools (i.e., email and text messages); iv) their views on the impact of social media use on service user's mental health and functioning; and v) their awareness of organisational guidelines around social media use in the workplace, with a view to make suggestions about how best to integrate social media use into clinical practice and maximise its benefits while minimising risks.

## Methods

Paper-based and online versions of the survey were disseminated from April 2018 to September 2020 in Mental Health Trusts in the Northwest of England and online via social media websites such as X (formerly Twitter). The online version of the survey used the REDCap^23,24^ platform. Eligible participants were staff members who had experience of working with individuals experiencing early psychosis and were currently employed in primary or secondary mental health services or in Third Sector organisations (e.g., charities). The study was approved by the West of Scotland Research Ethics Committee 4 (17/WS/0221). Written informed consent was provided by all participants.

The data reported in this paper is part of the broader eHealth and mHealth Interest Survey - Clinician Version study (EMIS-CV see Supplementary Table 1; Zhang et al., *in preparation*), which was developed to understand mental health professionals’ views on using digital health tools in clinical practice. In this paper, we report findings from the social media items included in this broader survey, which include questions about: i) staff's use of social media platforms; ii) their estimation of service user's use of social media platforms; iii) their communications with service users via social media platforms and other digital tools; iv) their views on the impact of social media use on service user's mental health and functioning; and v) their awareness of organisational guidelines around social media use in the workplace. The survey was designed by the research team based on findings obtained in previous focus groups with staff working in Early Intervention in Psychosis services (EIS).^25,26^ The survey was pilot tested with patient and public involvement and engagement (PPIE) contributors for acceptability and relevance. Staff working in both primary and secondary health care services were asked to complete either an online or paper-based version of the EMIS-CV. Quantitative data was analysed using R.^27^ Descriptive statistics were conducted to summarise the data.

## Results

A total of 155 staff completed the survey. A summary of participant demographic characteristics is presented in Table 1. The mean age of respondents was 39.7 years (SD = 10.35; range 19–63), and the mean time (years) working in current role was 5.6 years (SD = 6.63, range 0.05–30). The highest proportion of respondents were female (n = 119, 76.77%), White (n = 137, 88.39%), working in EIS (n = 62, 40%), and received postgraduate level education (n = 106, 68.39%). Participant job roles included: community psychiatric nurse (n = 32, 20.65%), care coordinator (n = 24, 15.48%) and psychologist (n = 21, 13.55%).

**Table 1.** Characteristics of survey respondents.

Characteristics	Values
**Age, mean (SD)**	
	39.73 (10.35)
**Gender, n (%)**	
Female	119 (76.77)
Male	36 (23.23)
**Ethnicity, n (%)**	
Asian or Asian British	11 (7.10)
Black, Black British, Caribbean or African	3 (1.94)
Mixed or multiple ethnic groups	2 (1.29)
Other ethnic group	1 (0.64)
White	137 (88.39)
Missing	1 (0.64)
**Service, n (%)**	
Charity sector	7 (4.52)
Community mental health team	10 (6.45)
Early intervention service	62 (40.00)
General practice	9 (5.80)
Home treatment team	41 (26.45)
Inpatient unit	6 (3.87)
Secondary care psychological services	13 (8.39)
Missing	7 (4.52)
**Education, n (%)**	
High school	3 (1.94)
College	4 (2.58)
Some university	5 (3.22)
University	36 (23.22)
Postgrad	106 (68.39)
Missing	1 (0.65)
**Job title, n (%)**	
Care coordinator	24 (15.48)
Support worker	10 (6.45)
Social worker	13 (8.39)
Community psychiatric nurse	32 (20.65)
Occupational therapist	7 (4.52)
Psychotherapist	12 (7.74)
Psychologist	21 (13.55)
Psychiatrist	11 (7.10)
GP	1 (0.64)
Other	23 (14.84)
Missing	1 (0.64)
**Years working in current role, mean (SD)**	
	5.62 (6.63)

**Figure 1.**
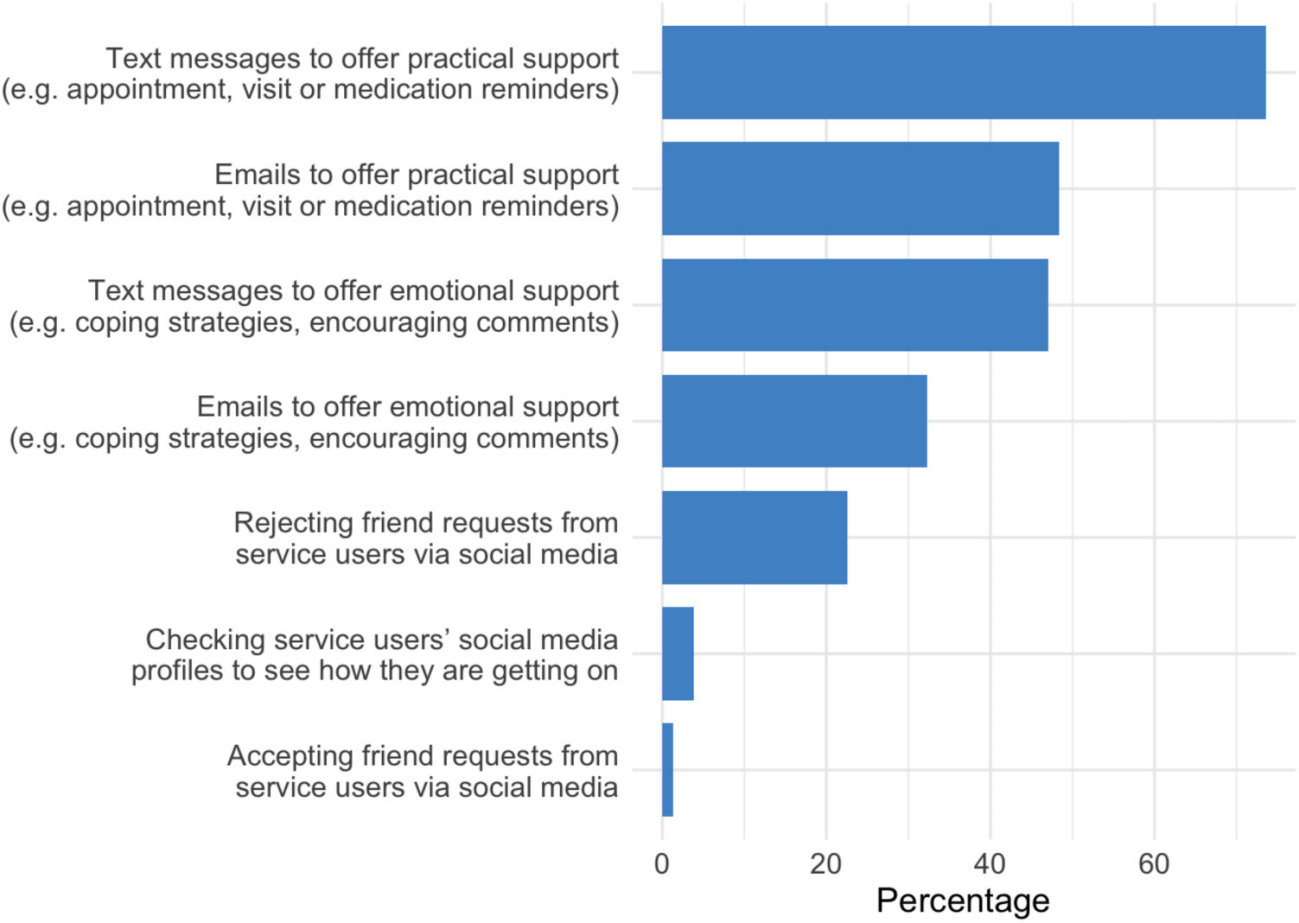
Experiences of staff communicating with service users via technology.

Regarding staff's usage of social media, of the 155 participants, more than three-quarters said they used a social media platform in their day-to-day life (n = 123/155, 79.36%). We asked participants to estimate the percentage of service users on their current caseload using a social media platform. A proportion of staff who responded to this item (n = 103/155, 66.45%) estimated that more than half of the service users on their caseload use social media in their day-to-day life, whereas a subgroup (n = 26/155, 16.77%) of staff reported that 100% of service users on their caseload use social media in their day-to-day life. Figure 1 shows participants’ reports about how they communicated with service users via technology. Of digital technologies available, staff most frequently use text messaging to offer ‘practical support’ (e.g., appointment, visit or medication reminders) to service users (n = 114/155, 73.55%), followed by emails (n = 75/155, 48.39%). In terms of offering ‘emotional support’ (e.g., coping strategies, encouraging comments), staff most frequently use text messages (n = 73/155, 47.10%), followed by emails (n = 50/155, 32.26%). 3.87% (n = 6/155) of staff respondents said they have checked service users’ social media profiles to see how the service user is getting on. 1.29% (n = 2/155) staff respondents reported that they have accepted friend requests from service users via social media, while nearly a quarter (n = 35/155, 22.58%) reported that they have rejected friend requests from service users via social media.

Most staff said that they were aware of NHS Trust guidelines around communicating with service users via technology (i.e., mobile phone and social media); however, a notable number of responders reported being either ‘not aware’ or ‘unsure’ about the guidelines (see Table 2). Specifically, nearly 70% (n = 107/155, 69.03%) and 60% (n = 93/155) staff said they were aware of Trust guidelines around communicating with service users using their personal mobile phone numbers and via text messages, respectively. While 61.29% (n = 95/155) staff were aware of guidelines around accepting friend requests from service users on social media, only half of responders (50.32%, n = 78/155) said that know the Trust guidelines about viewing service users’ social media profiles.

**Table 2.** Awareness of trust guidelines: n (%).

Guidelines	Yes	No	Unsure	Missing
Communicating with service users via text messages	93 (60)	20 (12.9)	33 (21.29)	9 (5.81)
Communicating with service users using personal mobile phone numbers	107 (69.03)	21 (13.55)	18 (11.61)	9 (5.81)
Viewing service users’ social media profiles	78 (50.32)	42 (27.1)	26 (16.77)	9 (5.81)
Accepting friend requests from service users on social media	95 (61.29)	32 (20.65)	17 (10.97)	11 (7.1)

Staffs’ attitudes towards general social media use are shown in Table 3. Most staff endorsed the item social media in general could benefit service users’ social functioning, with around 80% of responders reporting either ‘agree’ or ‘strongly agree’ to the statements ‘social media can help service users interact with friends and/or family’ (n = 128/155, 82.58%) and ‘using social media can help service users socialise with people’ (n = 123/155, 79.35%). Additionally, 47.74% (n = 74/155) endorsed the statement that engaging in a social media group with others with psychosis would benefit service users. However, participants also endorsed statements regarding the potential harms of using social media. 80.65% (n = 125/155) endorsed that social media makes people compare themselves with others. Over two thirds of respondents considered using media could exacerbate mental health symptoms, with 68.39% (n = 106/155), 63.23% (n = 98/155), and 36.13% (n = 56/155) reporting that they ‘agree’ or ‘strongly agree’ to the statements ‘social media contributes towards depression and/or anxiety’, ‘social media contributes towards paranoia or suspiciousness’, and ‘social media can make voices worse’, respectively.

**Table 3.** Staff attitudes towards social media: n (%).

Items	Strongly disagree	Disagree	Neutral	Agree	Strongly agree	Missing
Social media contributes towards depression and/or anxiety	1 (0.65)	4 (2.58)	28 (18.06)	70 (45.16)	36 (23.23)	16 (10.32)
Social media contributes towards paranoia or suspiciousness	–	8 (5.16)	33 (21.29)	71 (45.81)	27 (17.42)	16 (10.32)
Social media can make voices worse	1 (0.65)	12 (7.74)	70 (45.16)	47 (30.32)	9 (5.81)	16 (10.32)
Social media can help service users interact with friends and/or family	1 (0.65)	2 (1.29)	7 (4.52)	98 (63.23)	30 (19.35)	17 (10.97)
Using social media can help service users socialise with people	1 (0.65)	6 (3.87)	8 (5.16)	99 (63.87)	24 (15.48)	17 (10.97)
Using social media makes people compare themselves with others	1 (0.65)	1 (0.65)	11 (7.1)	75 (48.39)	50 (32.26)	17 (10.97)
It would be beneficial for service users to engage in a social media group with others with psychosis	2 (1.29)	3 (1.94)	60 (38.71)	66 (42.58)	8 (5.16)	16 (10.32)

## Discussion

We conducted a cross-sectional survey study to understand the mental health professionals’ views on social media use among people with psychosis. Staff expressed mixed attitudes toward using social media by endorsing statements about both benefits and negative effects. The foremost endorsed benefit was social media's potential to promote social interactions; the foremost concern was the potential for social media use to lead to harmful social comparisons. Additionally, most staff participants expressed concerns that using media could exacerbate mental health symptoms. The concerns may relate to the association between problematic use of social media and mental health.^28,29^ However, evidence indicates that most service users with psychosis use social media as part of daily life^30^ and view it as a favourable source for receiving mental health-related information or to elicit mental health-related support.^31^ It is therefore crucial to ensure staff hold a balanced views on service users’ use of social media, particularly to prevent negative attitudes from hindering service users from fully benefiting from the advantages that social media potentially offers.

We found relatively high social media platform usage among staff. Staff communicating with service users via text message and email was common; however, interacting with service users using social media platforms was not. This is consistent with previous survey studies showing that social media platforms were less frequently used for clinical purposes compared to other technologies.^32,33^ Furthermore, only 3.87% of staff in this study reported checking service users’ social media profiles to monitor their progress, a significantly lower percentage compared to a US study where 33.9% of staff had done so.^34^ This may be related to the finding that a notable number of staff reported that they were either unsure or unaware of specific NHS Trust guidelines regarding the use of digital technologies with service users, leading them to avoid using social media platforms for clinical purposes.

Based on these findings, managers should ensure staff are aware of organisational guidelines regarding utilising social media and other digital technologies to prevent ineffective or harmful practice. Well-established guidelines can improve staff clarity about their responsibilities when using social media in a work context.^35^ Additionally, staff training about up-to-date evidence on the relationship between social media use and its impact on mental health is needed to help guide clinicians in asking about online harms or behaviour during clinical assessment and/or when formulating a treatment plan*.*

One limitation of this study is that we were not able to collect information about how many potential participants were reached and subsequently did not take part in the survey, which limits our ability to assess the selection bias of this sample. In addition, staff members who participated may be more interested in social media compared to the wider group.

## Conclusions

Staff can play an important role in fully realising the potential of social media on mental health while mitigate its risks. While the mixed views expressed by staff about the use of social media platforms in psychosis may reflect the fact that existing evidence shows complex relationships between social media use and mental health, we need to ensure staff do not overestimate its risks or underestimate its benefits, so that they can offer tailored advice on social media use based on service users’ individual circumstances.

## Supplemental Material

sj-docx-1-dhj-10.1177_20552076251321059 - Supplemental material for Mental Health Staff's Views on Social Media Use Among People with Psychosis: A Cross-Sectional Survey Supplemental material, sj-docx-1-dhj-10.1177_20552076251321059 for Mental Health Staff's Views on Social Media Use Among People with Psychosis: A Cross-Sectional Survey by Xiaolong Zhang, Natalie Berry and Sandra Bucci in DIGITAL HEALTH
