# Supplementary material for: Mental Health Staff's Views on Social Media Use Among People with Psychosis: A Cross-Sectional Survey
Source: Digit Health. 2025 Mar 17;11:20552076251321059. doi: 10.1177/20552076251321059 (PMC11915250; doi:10.1177/20552076251321059)
Supplement: sj-docx-1-dhj-10.1177_20552076251321059 - Supplemental material for Mental Health Staff's Views on Social Media Use Among People with Psychosis: A Cross-Sectional Survey [file sj-docx-1-dhj-10.1177_20552076251321059.docx]

**Supplementary Table 1. EMIS-CV survey**

**Demographic information**

1. How old are you? ……..

2. What is your gender? ……………………

3. What is your ethnicity? ……………………

4. What service do you currently work in?

Early Intervention Service Community Mental Health Team

Home Treatment Team Secondary care psychological services

Charity sector Inpatient unit

General Practice

5. What is the highest level of education you have attended? Please tick to indicate

High school/Secondary school University (degree awarded)

College/Sixth form Postgraduate course

Some University

(degree not or not yet awarded)

6. What is your job title?

Care coordinator Support worker Social worker

Community Psychiatric Nurse Occupational therapist Psychotherapist

Psychologist Psychiatrist GP

Other - please state …………………………..

7. How long have you been working in your current role? (in years) ……………………………..

8. Have you ever referred a client to a research trial? (please tick to indicate)

Yes No

9. What factors would influence your decision to refer clients to a research trial? (please tick all that apply)

Evidence-base Professional past experience

Client risk Clients current prescription

Client preference Type of research

Clinical guidelines Resources/availability

MDT opinions Other (please state) …………………………….

**Technology ownership/use questions**

10. Which of the following do you use? (please circle to indicate):

| Mobile phone (personal) | Yes | No |
| --- | --- | --- |
| Mobile phone (work) | Yes | No |
| Smartphone (personal) | Yes | No |
| Smartphone (work) | Yes | No |
| Tablet computer (e.g. Ipad) (personal) | Yes | No |
| Tablet computer (e.g. Ipad) (work) | Yes | No |
| Laptop computer (personal) | Yes | No |
| Laptop computer (work) | Yes | No |
| Desktop computer (personal) | Yes | No |
| Desktop computer (work) | Yes | No |
| Internet | Yes | No |
| Social media | Yes | No |
| Smartwatch (e.g. Apple watch) | Yes | No |
| Fitness tracker (e.g. Fitbit) | Yes | No |

11. Please indicate how much you agree or disagree with the following statements? (please circle):

| Question | Strongly disagree | Disagree | Neither agree nor disagree | Agree | Strongly agree |
| --- | --- | --- | --- | --- | --- |
| I am enthusiastic about electronics and digital devices | 1 | 2 | 3 | 4 | 5 |
| I frequently look for new software or apps | 1 | 2 | 3 | 4 | 5 |
| My friends would describe me as “into” the latest technology | 1 | 2 | 3 | 4 | 5 |
| Technology could play a positive role in mental health services in the future | 1 | 2 | 3 | 4 | 5 |
| For me, technology is frustrating | 1 | 2 | 3 | 4 | 5 |

**Service users’ technology use/ownership**

12. Please provide an **estimate** of the proportion of your current caseload who own…: (please circle to indicate)

| A mobile phone | 0% | 1-24% | 25-49% | 50-74% | 75-99% | 100% |
| --- | --- | --- | --- | --- | --- | --- |
| A smartphone | 0% | 1-24% | 25-49% | 50-74% | 75-99% | 100% |
| A social media profile (e.g. Facebook, Twitter) | 0% | 1-24% | 25-49% | 50-74% | 75-99% | 100% |
| A tablet | 0% | 1-24% | 25-49% | 50-74% | 75-99% | 100% |
| A laptop computer | 0% | 1-24% | 25-49% | 50-74% | 75-99% | 100% |
| A desktop computer | 0% | 1-24% | 25-49% | 50-74% | 75-99% | 100% |
| A smartwatch (e.g. Apple Watch) | 0% | 1-24% | 25-49% | 50-74% | 75-99% | 100% |
| An activity tracker (e.g. FitBit) | 0% | 1-24% | 25-49% | 50-74% | 75-99% | 100% |

13. What barriers have you experienced service users with psychosis facing, if any, in being able to own or use a mobile phone? (please tick all that apply):

Struggling to afford mobile phones/smartphones Loss of mobile phones

Damage of mobile phones Technology use skills

Paranoia/suspiciousness about mobile phones Other (please state below)

Not applicable (I have not encountered any barriers) ………………………………..

14. Have you ever recommended an app to a service user to help with their physical health (e.g. stop smoking app, weight management app)? (please tick to indicate)

Yes No

15. Have you ever recommended an app to a service user to help with their mental health (e.g. mindfulness)

Yes No

16. Have you ever had the following experiences of communicating with service users via technology? (please circle to indicate)

| Text messages to offer practical support (e.g. appointment, visit or medication reminders) | Yes | No |
| --- | --- | --- |
| Text messages to offer emotional support (e.g. coping strategies, encouraging comments) | Yes | No |
| Emails to offer practical support (e.g. appointment, visit or medication reminders) | Yes | No |
| Emails to offer emotional support (e.g. coping strategies, encouraging comments) | Yes | No |
| Checking service users’ social media profiles to see how they are getting on | Yes | No |
| Accepting friend requests from service users via social media | Yes | No |
| Rejecting friend requests from service users via social media | Yes | No |

17. Are you aware of any Trust guidelines surrounding the following topics?

a. Communicating with service users via text messages:

Yes No Unsure

b. Communicating with service users using personal mobile phone numbers:

Yes No Unsure

c. Viewing service users’ social media profiles:

Yes No Unsure

d. Accepting friend requests from service users on social media:

Yes No Unsure

18. Have you ever recommended the following strategies to service users for self-management? (please circle to indicate)

| Tracking symptoms/experiences via a smartphone app | Yes | No (but I would consider recommending in the future) | No (and I wouldn’t consider recommending in the future) |
| --- | --- | --- | --- |
| Tracking symptoms/experiences via a website | Yes | No (but I would consider recommending in the future) | No (and I wouldn’t consider recommending in the future) |
| Tracking symptoms/experiences using a paper diary | Yes | No (but I would consider recommending in the future) | No (and I wouldn’t consider recommending in the future) |
| Listening to music or audio files via a smartphone to distract from voices or intrusive thoughts | Yes | No (but I would consider recommending in the future) | No (and I wouldn’t consider recommending in the future) |
| Recording auditory hallucinations via a smartphone | Yes | No (but I would consider recommending in the future) | No (and I wouldn’t consider recommending in the future) |
| Photographing visual hallucinations via a smartphone | Yes | No (but I would consider recommending in the future) | No (and I wouldn’t consider recommending in the future) |
| Setting alarms/reminders to help with medication management | Yes | No (but I would consider recommending in the future) | No (and I wouldn’t consider recommending in the future) |
| Using a calendar or set alarms/reminders for appointments | Yes | No (but I would consider recommending in the future) | No (and I wouldn’t consider recommending in the future) |
| Accessing an online forum to access support/connect with others | Yes | No (but I would consider recommending in the future) | No (and I wouldn’t consider recommending in the future) |

19. Have you ever used any of the following resources during appointments with service users? (please circle to indicate)

| Online written information about mental health | Yes | No (but I would consider doing this in the future) | No (and I wouldn’t consider doing this in the future) |
| --- | --- | --- | --- |
| Online videos (e.g. on YouTube) about mental health | Yes | No (but I would consider doing this in the future) | No (and I wouldn’t consider doing this in the future) |
| Smartphone camera to visually record formulations  Not applicable to my role | Yes | No (but I would consider doing this in the future) | No (and I wouldn’t consider doing this in the future) |
| Symptom monitoring information recorded by service users via a smartphone | Yes | No (but I would consider doing this in the future) | No (and I wouldn’t consider doing this in the future) |
| Symptom monitoring information recorded by service users via a website | Yes | No (but I would consider doing this in the future) | No (and I wouldn’t consider doing this in the future) |
| Symptom monitoring information recorded by service users using a paper diary | Yes | No (but I would consider doing this in the future) | No (and I wouldn’t consider doing this in the future) |
| A smartphone app to complete therapy tasks between sessions  Not applicable to my role | Yes | No (but I would consider doing this in the future) | No (and I wouldn’t consider doing this in the future) |
| A website to complete therapy tasks between sessions  Not applicable to my role | Yes | No (but I would consider doing this in the future) | No (and I wouldn’t consider doing this in the future) |
| Paper-based therapy tasks between sessions  Not applicable to my role | Yes | No (but I would consider doing this in the future) | No (and I wouldn’t consider doing this in the future) |

20. To what extent do you agree or disagree with the following statements? (please circle to indicate)

| Question | Strongly disagree | Disagree | Neutral | Agree | Strongly agree |
| --- | --- | --- | --- | --- | --- |
| Social media contributes towards depression and/or anxiety | 1 | 2 | 3 | 4 | 5 |
| Social media contributes towards paranoia or suspiciousness | 1 | 2 | 3 | 4 | 5 |
| Social media can make voices worse | 1 | 2 | 3 | 4 | 5 |
| Social media can help service users interact with friends and/or family | 1 | 2 | 3 | 4 | 5 |
| Using social media can help service users socialise with people | 1 | 2 | 3 | 4 | 5 |
| Using social media makes people compare themselves with others | 1 | 2 | 3 | 4 | 5 |
| It would be beneficial for service users to engage in a social media group with others with psychosis | 1 | 2 | 3 | 4 | 5 |

21. Would you want to receive the symptom information (e.g. anxiety, depression, hallucinations) service users have entered into a mobile phone app? (please tick to indicate)

Yes (I would want the information to be automatically transferred to the team)

Yes (I want to receive the information, but I would want service users to take it to appointments themselves to show me, rather than have data automatically transferred)

No (I would not want to see the symptom information service users enter on an app)

22. If you were told about a new trial implementing a smartphone app informed by the principles of CBT to help people with psychosis self-manage their mental health, do you think you would refer service users to receive it? (please tick to indicate)

Yes No Unsure

Please could you provide the reason(s) for the answer you provided above:

………………………………………………………………………………………………………………………………………………………………………………………………………………………………………………………………………………………………………………………………………………………………………

23. To what extent do you agree or disagree (1 = strongly disagree; 5 = strongly agree) that the following barriers would affect the likelihood of you referring service users to receive a mental health app? (please tick all that apply)

| Barrier | Strongly disagree | Disagree | Neutral | Agree | Strongly agree |
| --- | --- | --- | --- | --- | --- |
| Smartphone handset and data costs | 1 | 2 | 3 | 4 | 5 |
| Service users’ technology and/or literacy skills | 1 | 2 | 3 | 4 | 5 |
| My own technology skills and knowledge |  |  |  |  |  |
| Poor motivation to engage with an app | 1 | 2 | 3 | 4 | 5 |
| Concerns about how helpful a mental health app would be | 1 | 2 | 3 | 4 | 5 |
| Concerns a mental health app would be used as an excuse to replace face-to-face support | 1 | 2 | 3 | 4 | 5 |
| Privacy/confidentiality of data inputted | 1 | 2 | 3 | 4 | 5 |
| Service users feeling suspicious or paranoid about using smartphones and apps | 1 | 2 | 3 | 4 | 5 |
| Concerns about identifying risk | 1 | 2 | 3 | 4 | 5 |
| Apps will miss the therapeutic relationship | 1 | 2 | 3 | 4 | 5 |

24. Please list any other concerns that you can think of that would prevent you from referring a service user to a trial providing a CBT-informed smartphone app.

………………………………………………………………………………………………………………………………………………………………………………………………………………………………………………

25. To what extent do you agree or disagree (1 = strongly disagree; 5 = strongly agree) that the following are potential advantages of mental health apps? (please tick all that apply)

|  | Strongly disagree | Disagree | Neutral | Agree | Strongly agree |
| --- | --- | --- | --- | --- | --- |
| Ability to access an app at any time and in any location | 1 | 2 | 3 | 4 | 5 |
| Opportunity to take control over mental health needs | 1 | 2 | 3 | 4 | 5 |
| Opportunity to increase understanding about symptoms and experiences | 1 | 2 | 3 | 4 | 5 |
| Apps can be anonymous | 1 | 2 | 3 | 4 | 5 |
| Opportunity for service users to take up-to-date records of symptoms and experiences to clinicians | 1 | 2 | 3 | 4 | 5 |
| Opportunity to identify symptom triggers and patterns | 1 | 2 | 3 | 4 | 5 |
| Cost-effective alternative to face-to-face support | 1 | 2 | 3 | 4 | 5 |
| Potential for staff to intervene early if increases in symptoms are identified | 1 | 2 | 3 | 4 | 5 |
| Increased privacy in comparison to paper-based monitoring or tasks | 1 | 2 | 3 | 4 | 5 |
| Having a mental health app available is normalising and de-stigmatising | 1 | 2 | 3 | 4 | 5 |

26. Please list any other advantages of using mental health apps

………………………………………………………………………………………………………………………………………………………………………………………………………………………………………………………………………………………………………………………………………………………………………
